# Predictions built upon belongings

**DOI:** 10.3389/fpsyg.2022.994098

**Published:** 2022-10-24

**Authors:** Luigi Grisoni

**Affiliations:** ^1^Brain Language Laboratory, Department of Philosophy and Humanities, Freie Universität Berlin, Berlin, Germany; ^2^Cluster of Excellence “Matters of Activity. Image Space Material”, Humboldt Universität zu Berlin, Berlin, Germany

**Keywords:** semantic processing, predictive processing, Prediction Potential (PP), N400, cell assembly

The ability to accurately predict possible continuations of contexts leads to relatively effortless processing of the predicted (i.e., pre-activated) item both in perceptual (Garrido et al., 2009; Lieder et al., 2013; Grisoni et al., 2019a,b) and language tasks (Grisoni et al., 2017, 2020; Pickering and Gambi, 2018; Grisoni and Pulvermüller, 2022).

In the most ideal case, preactivations concern one specific stimulus (e.g., a word), so that, before its actual presentation, there is an increase of activation in the circuit representing the expected stimulus (prediction). When the presented stimulus matches the preactivations, it brings less novel, unprocessed, information as compared to the case in which a stimulus was unexpected overall, a pattern reminiscent of priming (McNamara, 2005; Grisoni et al., 2016). In its simplicity these considerations open up some not-trivial problems, such as: is it possible to distinguish between *genuinely* context-related (e.g., sentence fragment) and *genuinely* expected-stimulus-related activations? Or, alternatively, which type of information is predicted? Here the issue is: if a specific stimulus (e.g., the eagle) is expected in a specific context (e.g., “The emblem of Germany is the …”), it must be for some relevant context and stimulus-related information. In other words, stimulus predictability is not *only*^1^,^1^ an *intrinsic* feature of any stimulus, but rather it emerges from the specific relationship that the stimulus entertains with the context constraining its expectation; in this sense, any stimulus could be either predictable or unpredictable, depending on the preceding context.

To possibly answer one of the aforementioned questions (i.e., which type of semantic information is predicted?) one might start to investigate whether the entire distributed circuit (distributed cell assemblies, see below, Pulvermüller, 1999; Pulvermüller and Fadiga, 2010) representing a word (Whole-item hypothesis) is called into play during semantic predictions, or, alternatively, whether it is limited to some specific sub-components (Partial-item hypothesis). One possibility is that predictable sentence fragments, but not unpredictable ones, *somehow* trigger the selection, and hence the pre-activation, of a specific word from the lexicon, and therefore ignites the entire cortical circuit representing the selected item (e.g., phonological, and semantic levels involved). Alternatively, predictive processing might involve only those semantic, perceptual features used to complete the preceding sentence fragment. In this case, a word is predictable in a specific context insofar as all the necessary semantic features (e.g., motor, sensory, etc.) used to continue the preceding fragment belong to the distributed cell assembly (CA, Pulvermüller and Preibl, 1991; Pulvermüller and Fadiga, 2010; Pulvermüller, 2018) representing that word. These features are codified just by a subset of all the *neurons* belonging to this specific CA, and they form the Set of Belonging (SB), meaning the set containing the nerve cells that codify the specific semantic information necessary to complete the preceding context (e.g., sentence fragment). Therefore, the SB of a specific word (e.g., *write*) might differentially overlap with the long-term memory trace of that word (i.e., the whole CA), and the specific SB topography and extension might crucially depend on the specific environment of constraint preceding the final word. For example, a fragment like “I go to the blackboard and I…” might induce SB pre-activations which (slightly) differ in topography from the SB preactivated by another predictable context constraining the expectation of the same word *write*, such as “I take the pen and I….” To complete the former fragment, specific features belonging to a specific instantiation of the word *write* are foregrounded (e.g., writing takes place on a vertical plane, while the agent stands in front of the blackboard producing a specific sound etc…); whereas in the second case different features are foregrounded (e.g., writing takes place on the horizontal plane, while the agent is seated producing different, or no, sounds).

Furthermore, according to the Partial-item hypothesis, predictable fragments could be completable with features belonging to either one or more CAs. If the SB overlaps with just one CA, a fragment is predictable with low entropy (i.e., just one word is predicted); vice versa, if the SB overlaps with two or more CAs, that is, if the set of features necessary to complete a sentence fragment belong to more than one CA, the context is predictable with a higher entropy. Fragments like “I pet my…” might constraint the expectation of at least two possible continuations (“cat” and/or “dog”) because the relevant features (i.e., the fact that it is a pet, with fur, etc…) belong equally to these two words and, therefore, the SB overlaps with these two CAs. Finally, if no CA contains all the relevant features to complete the sentence fragment, that is, if the SB is not entirely a subset of any specific CA, then the fragment is, to some extent, unpredictable. In this case, the completion relies on features which are broadly distributed in the cortical tissue and weakly interconnected with each other as they might belong to different circuits. The result is thus weaker and more widespread brain responses recorded on the scalp.

Recently, it has consistently been shown (Grisoni et al., 2020) that when German participants are presented with high-cloze (HC), predictable sentence fragments (e.g., “The emblem of Germany is the … [eagle]”), a slow-wave known as the Prediction Potential (PP, Pulvermüller and Grisoni, 2020) emerged before the final word presentation (Figure 1A); whereas matched sentence fragments which do not strongly predict a unique subsequent word (low-cloze, LC, sentences, e.g., “The emblem of my family is the … [eagle]”) elicited relatively smaller PPs prior to, but enlarged N400 responses following the critical word (Figure 1A). The observation of linear functional relationships between the PP mean amplitudes and the co-occurrence frequencies of the final target word (e.g., eagle) with the preceding context (e.g., “The emblem of Germany is the …”) suggest that anticipatory mechanisms rely on the specific relationship between the final stimulus and the preceding context, rather than on intrinsic features of the expected stimulus (see above, and Figure 1B). The observation of negative correlations, at different channel locations, between the PP and the subsequent post-stimulus, N400, brain responses (Figure 1C) is consistent with the idea of a functional relationship between these two brain signals and with both the Whole- and Partial-item hypotheses. Indeed, both these hypotheses suggest that the inverse, functional, relationship between the PP and the N400 could be explained as a difference in the activity preceding word onset, a mechanism reminiscent of priming (see above). It remains to ascertain whether this priming-like effect operates on the whole cortical representation of the word (i.e., full ignition of the circuit representing the expected word) or just on a portion of the circuit representing the expected item (i.e., partial ignition of the circuit). One way of testing these two hypotheses against each other is to look at the source estimations which, despite the well-known limitations in relation to spatial resolution (Helmholtz, 1853; Hauk et al., 2011), might nevertheless return important indications which would implicate either the Whole- or the Partial-item hypotheses, as they are expected to differ in brain areas further away from each other (see below).

**Figure 1:**
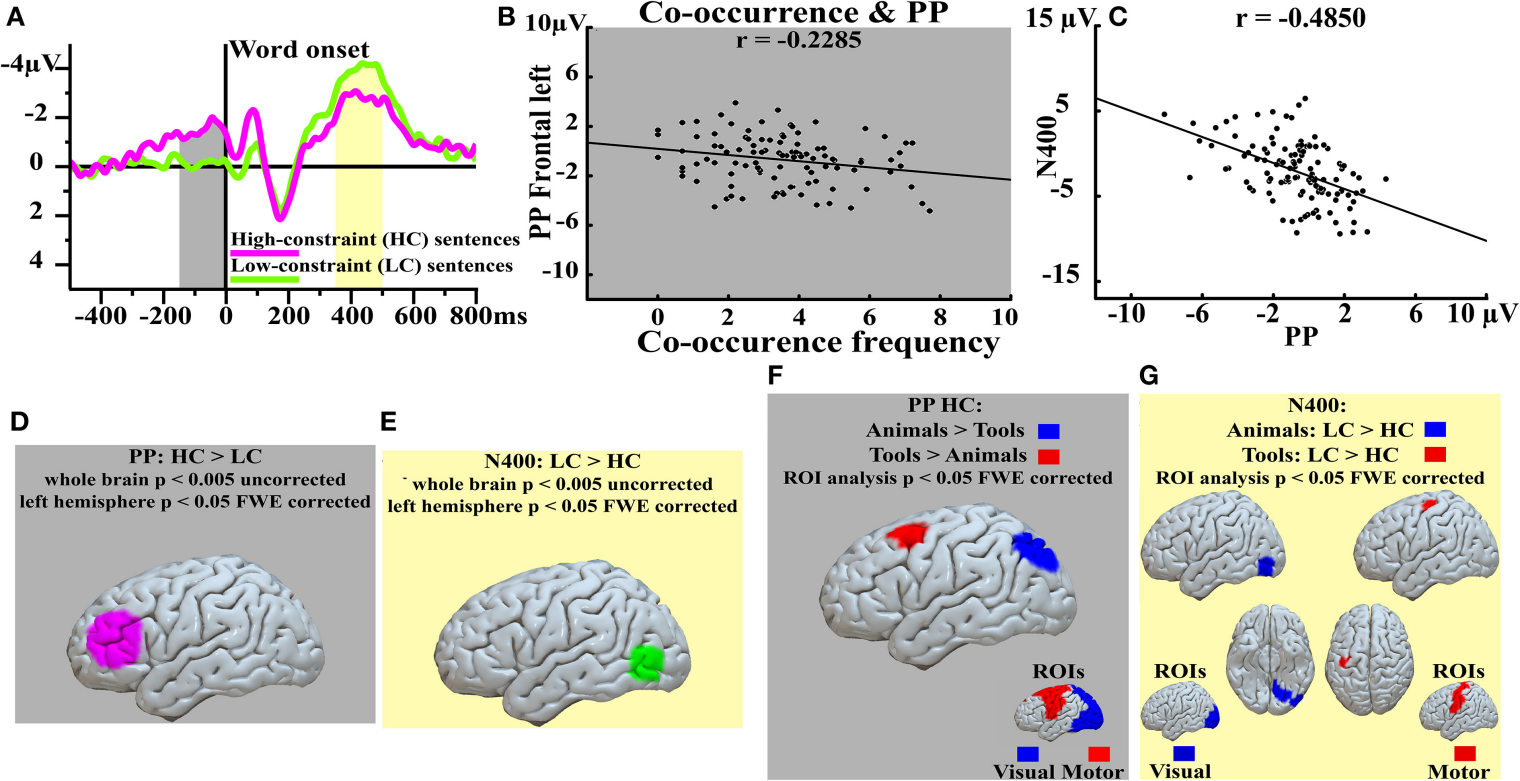
Event-related potentials (ERPs) and source analysis results. Top left **(A)** ERPs elicited by predictable (HC, magenta) and unpredictable (LC, green) sentences at both the pre- (PP, gray window) and post-stimulus (N400, yellow window) latencies. Top central **(B)** significant correlation between the corpus-based, co-occurrence frequency (x-axis) and the PP (y-axis) responses at left-frontal electrodes. Top right **(C)** negative correlation between the PP (x-axis) and the N400 (y-axis) responses recorded at centro-occipital electrodes. Bottom left **(D)** and bottom central **(E)** sources contrasts at the PP latency [HC > LC, **(D)**] and at the N400 latency [LC > HC, **(E)**]. Sub-panel **(F)** shows the contrasts of the source estimations (Region of Interest, ROIs, analysis; the ROIs are depicted at the bottom right of this sub-panel) HC: Tools > Animals (red) and HC: Animals > Tools (blue) at the pre-stimulus, PP, latency; whereas sub-panel **(G)** shows the contrasts of the source estimations (Region of Interest, ROIs, analysis; the ROIs are depicted at the bottom right of this sub-panel) Tools: LC > HC (red) and Animals: LC > HC (blue) at the post-stimulus, N400, latency. Modified from (Grisoni et al., 2020).

The main PP and N400 cortical generators were observed in inferior prefrontal (Figure 1D) and in posterior temporal areas (Figure 1E), respectively, whereas semantic category-specific cortical generators at both pre- and post-word latencies emerged in posterior, visual brain areas for animal nouns (e.g., eagle) but in prefrontal, motor regions for action-related, tool, nouns (e.g., horn) (Figures 1F,G Region of Interest, ROI, analysis). When a tool noun, such as “hammer,” was strongly predicted by context, relatively enhanced activation in motor and premotor areas was observed, whereas predicting an animal word, such as “rabbit,” was associated with relatively enhanced predictive activation in posterior, parieto-occipital visual areas. A similar dissociation had already been reported after word presentation (Martin et al., 1996; Kiefer, 2001; Carota et al., 2012, 2017; Kiefer and Pulvermüller, 2012) and could be replicated in this study also at the subsequent N400 latency. Overall, the observation of cluster of activations also in specific modality preferential brain areas are in line with those neurocognitive and neurolinguistic models for which words are stored by means of cell assemblies (CAs) distributed in specific modality preferential and multimodal cortical areas depending on the meaning of the word (Pulvermüller and Fadiga, 2010). According to these models, words are stored by means of representation units distributed across perisylvian cortical areas where neurons codify the specific auditory and articulatory profile of the word (phonological level), and in modality preferential and multimodal brain areas where *semantic neurons* codify the semantic level (*semantic neurons* in, for example, prefrontal, motor, or posterior-ventral, visual, regions, for action or visually related concrete words, respectively). The source estimations presented above are, therefore, more consistent with the Partial-item hypothesis; indeed, if the Whole-item hypothesis were correct, PP source estimations should have shown clusters of activity also in word form, perisylvian, areas; conversely, the observation of clusters of activity in lateral prefrontal areas (for the contrast HC > LC) (Figure 1D) and in specific modality preferential brain areas depending on the semantic category of the expected noun (i.e., prefrontal, motor, for tool nouns; but posterior, ventral for animal nouns) (Figure 1F) but not in perisylvian (i.e., neither in articulatory, motor ventral; nor in auditory, superior temporal) areas is overall more consistent with the Partial-item hypothesis, which assumes predictive activity in specific semantic sub-components (i.e., SBs) of the distributed representation units. The Partial- and Whole-item hypothesis also predicts different patterns of activation at post-word, N400, latencies. Indeed, if the pre-activations involved both the phonological and the semantic levels (Whole-item hypothesis), then perisylvian activity should be greater in LC than HC at these latencies due to a difference of activity within these areas before a word appears. Instead, the observation of clusters of activity in modality preferential brain regions, but not in perisylvian areas, at the N400 latency is consistent with the hypothesis that these areas were equally (in)activated (before) after word presentation in the two contexts (Figures 1E,G). Finally, the observation that the N400 was also manifest in HC sentences, albeit smaller than the N400 elicited by the LC fragments, is consistent with the assumption that only a portion (i.e., the SB) of the relevant CA was preactivated before word onset and, when the word is finally perceived, the remaining portion of the CA (in perisylvian, multimodal, and modality preferential brain areas) ignites thus eliciting yet another reliable post stimulus response (Figure 1A).

The observation that lateral prefrontal areas are particularly important in predictive processing (see Figure 1D and also, Fuster and Bressler, 2015; Grisoni et al., 2017; Leon-Cabrera et al., 2019; Grisoni and Pulvermüller, 2022) has particular significance within the Partial-item hypothesis. Indeed, it is well-known that these regions are densely connected with both sensory and motor associative areas (Fuster, 2001; Fuster and Bressler, 2015) and, therefore, these regions could act as a predictive hub targeting the relevant *semantic neurons* which allow specific belongings of the expected word within the preceding context; an interpretation which also matches previous accounts according to which the core functions of the lateral prefrontal cortex is the integration of linguistic information at several levels (e.g., phonological, semantic) of language processing (for example Hagoort, 2005). In this framework, therefore, the SB might represent an attempt to solve semantic integration, that is, how to integrate a word within a previous context. However, although some preliminary evidence seems to support the Partial-item hypothesis here presented, I acknowledge that more research is necessary to test this hypothesis and to come to a firm conclusion.

To conclude, predictive processing could help elucidate how concepts are integrated into wider contexts (i.e., sentence, discourse) and, in this sense, predictive processing might fulfill one of the most of its crucial functions, namely, reducing much of the ambiguity inherent in most linguistic utterances.

## Author contributions

LG conceptualized and wrote this manuscript.

## Funding

This work was supported by the Deutsche Forschungsgemeinschaft (DFG) (PhoNet; Pu 97/25-1). I acknowledge support by the Open Access Publication Initiative of Freie Universität Berlin.

## Conflict of interest

The author declares that the research was conducted in the absence of any commercial or financial relationships that could be construed as a potential conflict of interest.

## Publisher's note

All claims expressed in this article are solely those of the authors and do not necessarily represent those of their affiliated organizations, or those of the publisher, the editors and the reviewers. Any product that may be evaluated in this article, or claim that may be made by its manufacturer, is not guaranteed or endorsed by the publisher.

## Footnotes

^1^Absolute word frequency might be considered a measure of the intrinsic predictability of a word in any context, however, co-occurrence frequency, or conditional probability, is what can explain the expectation of a specific stimulus in a specific context.
